# Ultrasound Measurement of Foetal Kidney Length during Healthy Pregnancy: Relationship with Gestational Age

**DOI:** 10.4314/ejhs.v33i1.13

**Published:** 2023-01

**Authors:** Enefia K Kiridi, Peter C Oriji, Datonye C Briggs, Johnpatrick U Ugwoegbu, Chioma Okechukwu, Adedotun D Adesina, Akaninyene E Ubom, Panebi Y Bosrotsi, Abednigo O Addah, Isaac J Abasi

**Affiliations:** 1 Department of Radiology, Niger Delta University Teaching Hospital, Okolobiri, Nigeria; 2 Silhouette Radiodiagnostic Consultants, Yenagoa, Bayelsa State, Nigeria; 3 Department of Obstetrics and Gynaecology, Federal Medical Centre, Yenagoa, Nigeria; 4 Department of Paediatrics and Child Health, Rivers State University Teaching Hospital, Port Harcourt, Nigeria; 5 Department of Radiology, Federal Medical Centre, Owerri, Nigeria; 6 Department of Medical Services, Nigerian Law School, Yenagoa Campus, Yenagoa, Nigeria; 7 Oasis Public Health Consulting Ltd, Yenagoa, Nigeria; 8 Department of Obstetrics, Gynaecology and Perinatology, Obafemi Awolowo University Teaching Hospitals Complex, Ile-Ife, Nigeria; 9 International Federation of Gynaecology and Obstetrics (FIGO) Committee on Childbirth and Postpartum Haemorrhage; 10 Department of Obstetrics and Gynaecology, Diete Koki Memorial Hospital, Yenagoa, Nigeria; 11 Department of Obstetrics and Gynaecology, Niger Delta University Teaching Hospital, Okolobiri, Nigeria

**Keywords:** Foetal kidney length, Normal pregnancy, Gestational age

## Abstract

**Background:**

Foetal kidney length (FKL) measurements and comparisons to normal charts can be used to assess the development of the foetal kidneys throughout the entire course of pregnancy. This study was designed to assess FKL between 20 — 40 weeks' gestation, establish reference ranges for FKL and determine the relationship between FKL and gestational age (GA) in normal pregnancy.

**Methods:**

This descriptive, cross-sectional study was conducted between March-August 2022, at the Obstetric Units and Radiology Departments of the two tertiary health facilities, one secondary facility and one radio-diagnostic facility in Bayelsa State, Southern Nigeria. Transabdominal ultrasound scan was used to evaluate the foetal kidneys. The relationship between foetal kidney dimensions and GA was explored using Pearson's correlation analysis. Linear regression analysis was done to define the relationship between GA and mean kidney length (MKL). A nomogram predicting GA from MKL was constructed. Level of significance was set at p<0.05.

**Results:**

There was a very strong significant correlation between foetal renal dimensions and GA. The correlation coefficient between GA and mean FKL, width and anteroposterior diameter were 0.89 (p=0.001), 0.87 (p=0.001) and 0.82 (p=0.001), respectively. A unit change in mean FKL corresponded to a 79% change in GA (ɼ2), showing a very strong association between mean FKL and GA. The regression equation: GA = 9.87 + 5.91 x MKL, was derived for estimation of GA for a given MKL.

**Conclusions:**

Our study revealed a significant relationship between FKL and GA. The FKL can therefore be reliably used to estimate GA.

## Introduction

The definitive foetal kidney begins to develop in the first trimester as a fusion of the ureteric bud and metanephric mesoderm. Development of the kidney begins at seven weeks, and by 11 weeks' gestation, the kidney begins to function properly. As early as 9 to 12 weeks' gestation, prenatal ultrasonography (USS) can detect the foetal kidneys, which have a lobular appearance and are located in the paraspinal area (1). The average length of the kidney is about 1 cm at 12 weeks, and 2.7 cm at 20 weeks (1). Corticomedullary differentiation takes place between 15 and 20 weeks, and by 20 weeks, an echogenic cortex with the hypoechogenic, dark renal pyramids of the medulla should be seen (1).

Renal length measurements and comparisons to normal charts can be used to assess the development of the foetal kidneys throughout the entire course of pregnancy (2–4). The ultrasonogram is regarded as a good tool for evaluating growth and anomalies associated with the foetal kidneys and can aid in the early diagnosis and treatment of some kidney-related abnormalities. Nevertheless, several ultrasound-based studies have demonstrated that beyond the detection of renal anomalies, a relationship exists between foetal kidney length (FKL) and gestational age of the developing foetus and should be considered when estimating the duration of pregnancy (3–5).

There are a number of surrogate measurements such as gestational sac (GS), crown-rump length (CRL), biparietal diameter (BPD), head circumference (HC), femur length (FL) and abdominal circumference (AC), used for estimating the GA of the foetus. However, none has been reported to be the most accurate or reliable for estimating GA. In fact, Benson and Doubilet reported that the accuracy of ultrasound foetal biometric measurements decreases as pregnancy advances (6) Other authors (7,8) have suggested that using a combination of these surrogate foetal measurements is more accurate than using each in isolation, and yet, others (5,9) have opined that FKL appears to be a better measure for GA. So, even if it is clear that sonography of foetal kidney size is of benefit for detecting foetal kidney abnormalities, its use as a potential ‘stand-alone’ measure to estimate GA of pregnancy, especially when dates are unsure, needs further evaluation (5,9,10).

The need for an accurate estimation of gestational age, especially in a high-risk pregnancy for obstetricians and paediatricians alike, cannot be overemphasized. It will enable adequate and comprehensive care to ensure safe maternal and foetal outcomes. Transvaginal sonography, which may be used between 14 and 17 weeks of gestation, and transabdominal ultrasound scan USS, which can be used from 18 weeks of gestation and upwards, are both reliable methods for measuring the foetal kidney (2).

Literature on the relationship between FKL and GA of pregnancy is sparse in our setting. This study therefore sought to assess FKL between 20 – 40 weeks' gestation, establish reference ranges for FKL dimensions, and determine the relationship between FKL and GA in normal pregnancy among a sample of pregnant women in Southern Nigeria.

## Materials and Methods

**Study setting:** This descriptive, cross-sectional study was conducted at the Obstetric Units and Radiology Departments of the Federal Medical Centre, Yenagoa, Niger Delta University Teaching Hospital, Okolobiri, Diete Koki Memorial Hospital, Yenagoa, and Silhouette Radio-diagnostic Consultants, Yenagoa, all in Bayelsa State, South-South Nigeria. The first two study centres are tertiary health facilities that provide specialised gynaecological services to women in Bayelsa State and serve as referral centres for other hospitals in Bayelsa State, and surrounding Rivers and Delta States, both in South-South Nigeria. The third study centre is a secondary health institution. The fourth study centre is the biggest Radio-diagnostic Institution in Bayelsa State, Nigeria. The study was conducted from March – August 2022.

**Ethics**: Ethical approval for this study was obtained from the Research and Ethics Committee of the Federal Medical Centre, Yenagoa, Bayelsa State, Nigeria (FMCY/REC/ECC/2022/628).

**Sample size:** The sample size of 423 for this study was calculated using the Fisher's formula (11). All consenting women with normal singleton pregnancy between 20 — 40 weeks' GA were recruited for the study. Women with multiple gestation, medical conditions in pregnancy, foetal congenital or amniotic fluid abnormalities, intrauterine foetal growth restriction, foetal renal structural or functional abnormalities, indistinct renal borders on USS, and those who smoke or are unsure of their last menstrual period were excluded from the study.

**Study recruitment**: Four hundred and twenty-three consecutive consenting, eligible women were recruited for this study. The women were recruited from the antenatal clinics of the study centres.

Women who met the inclusion criteria for the study were counselled, and after obtaining written informed consent, were enrolled. A structured questionnaire was used to obtain and document the study participants' sociodemographic characteristics and other clinical information. Gestational age was calculated from the last menstrual period, which was correlated with the GA from an early USS. The women were thereafter referred to the Radiology Departments of the study centres for USS evaluation of the foetus and foetal kidneys.

**Procedure**: Ultrasound scans were performed transabdominally, and were performed by four Consultant Radiologists (one for each centre), with special interest and expertise in foetal renal scans. With the patient lying supine, and the abdomen and pelvis exposed, adequate ultrasound gel was applied to the lower anterior abdominal wall/pelvis. The gel served to remove air from the skin, and for ease of transducer movement. A real-time, grayscale, 3.5 MHz curvilinear array transducer of a 2012 Philips HD11 ultrasound machine was used to obtain measurements of all foetal biometric parameters. The probe was moved back and forth on the skin, and in orthogonal planes, with gain adjusted, as required, for good image quality. Estimated gestational age, foetal biometric parameters and estimated foetal weight (EFWT) were determined using the Hadlock's method, which included foetal BPD, HC, FL and AC measurements taken at specific reference points (12).

The right and left foetal kidneys were visualised using the superiorly placed liver and stomach respectively as land marks. The longitudinal axis of each kidney was measured by orientating the probe through 90° on either side of the abdominal aorta. The largest longitudinal image of the superior and inferior outer poles of each kidney was obtained and frozen on the monitor screen. The kidney length was measured from the superior outer pole to the inferior outer pole, using electronic calipers. To reduce inter- and intraobserver error, three measurements were obtained for each kidney, and the mean value (in millimeters) was recorded. The adrenal glands were excluded from these measurements.

**Data analysis**: Data from the study proforma was checked daily for completeness and correctness, and were entered directly into a statistical software, Statistical Product and Service Solutions version 25.0 (SPSS Inc., Chicago, IL, USA), which was used for data cleaning and analysis. Categorical variables (parity and age-group) were summarised using frequencies and percentages, while continuous variables (maternal height and weight, estimated gestational age, kidney length, width, and anteroposterior diameter) were summarised using mean and standard deviation after a normality (Shapiro-Wilk) test revealed that the variables were normally distributed. The mean kidney dimensions were calculated by dividing the sum of the right and left kidney dimensions by 2. The relationship between the kidney dimensions and estimated gestational age, maternal height and weight was explored using the Pearson's correlation analysis. The mean kidney length had the strongest correlation with the estimated gestational age; hence, it was used in a logistic regression analysis in defining the relationship between estimated gestational age and mean kidney dimension. The equation was thereafter used in predicting gestational ages at different kidney lengths, thereby, producing a nomogram. Level of significance was set at p<0.05.

## Results

**Sociodemographic and obstetric characteristics of study participants**: The mean age of the women was 29.03 ± 6.35 years. The median parity was 1, with a range of 0 to 7. Mean body mass index and gestational age were 27.75 ± 4.12 kg/m^2^ and 31.8 ± 5.5 weeks, respectively (Table 1).

**Table 1:** Maternal sociodemographic and obstetric characteristics

Characteristics	Frequency (n = 423)	Percent
**Age (years)**		
<20	24	5.7
20 – 24	85	20.1
25 – 29	136	32.2
30 – 34	105	24.8
>35	73	17.3
**Age range in years**	16 – 40	
**Mean age ± SD in years**	29.4 ± 5.6	
**Weight in kg (mean ± SD)**	71.67 ± 9.08	
**Height in metres (mean ± SD)**	1.59 ± 0.63	
**Parity**		
Nulliparity	170	40.2
Primiparity	107	25.3
Multiparity	109	25.8
Grand-multiparity	37	8.7
**Median parity (range)**	1 (0 – 8)	
**GA (weeks)**		
20 – 24	99	23.4
25 – 28	82	19.4
29 – 32	81	19.1
33 – 36	81	19.1
37 – 40	80	18.9
**Gestational age in weeks (mean ± SD)**	30.79 ± 4.28	

SD = Standard Deviation.

**Foetal kidney dimensions**: The mean right and left kidney lengths were 2.40 ± 0.32 cm and 2.20 ± 0.06 cm, respectively, at 20 weeks' gestation; and 4.75 ± 0.15 cm and 5.00 ± 0.45 cm, respectively, at 40 weeks' gestation. The mean kidney anteroposterior diameter and kidney width were 1.89 ± 0.32 cm and 2.24 ± 0.47 cm, respectively. The mean kidney length ranged from 2.30 ± 0.10 cm at 20 weeks' gestation to 3.70 ± 0.82 cm at 40 weeks, while the mean anteroposterior diameter was 1.15 ± 0.08 cm at 20 weeks and 1.89 ± 0.32cm at 40 weeks' gestation. The mean kidney width followed the same trend, increasing from 1.30 ± 0.07 cm at 20 weeks to 2.24 ± 0.47 cm at 40 weeks' gestation (Table 2).

**Table 2:** Right, left and mean foetal kidney dimensions at different GA among the study participants

GA in weeks	*Freq	Right kidney dimensions in cm			Left kidney dimensions in cm			Mean kidney dimensions in cm		
		
		Length	¶AP Diameter	Width	Length	¶AP Diameter	Width	Length	¶AP Diameter	Width
20	19	2.40 ± 0.32	1.00 ± 0.08	1.40 ± 0.07	2.20 ± 0.16	1.30 ± 0.09	1.20 ± 0.07	2.30 ± 0.10	1.15 ± 0.08	1.30 ± 0.07
21	20	2.50 ± 0.20	1.10 ± 0.07	1.50 ± 0.09	2.30 ± 0.23	1.40 ± 0.08	1.30 ± 0.09	2.40 ± 0.20	1.25 ± 0.07	1.40 ± 0.09
22	19	2.60 ± 0.14	1.20 ± 0.08	1.60 ± 0.11	2.40 ± 0.25	1.50 ± 0.06	1.40 ± 0.11	2.50 ± 0.13	1.35 ± 0.15	1.50 ± 0.11
23	20	2.70 ± 0.16	1.30 ± 0.07	1.70 ± 0.08	2.50 ± 0.18	1.60 ± 0.18	1.50 ± 0.16	2.60 ± 0.17	1.45 ± 0.17	1.60 ± 0.16
24	21	2.85 ± 0.05	1.50 ± 0.10	1.90 ± 0.10	2.70 ± 0.11	1.80 ± 0.10	1.65 ± 0.05	2.78 ± 0.08	1.65 ± 0.10	1.78 ± 0.08
25	20	2.90 ± 0.07	1.50 ± 0.11	1.91 ± 0.09	2.70 ± 0.07	1.80 ± 0.11	1.70 ± 0.06	2.80 ± 0.12	1.75 ± 0.09	1.85 ± 0.09
26	20	2.90 ± 0.08	1.60 ± 0.03	2.00 ± 0.04	2.80 ± 0.09	1.90 ± 0.10	1.70 ± 0.07	2.85 ± 0.08	1.75 ± 0.11	1.85 ± 0.10
27	20	2.90 ± 0.09	1.50 ± 0.04	2.10 ± 0.05	2.80 ± 0.08	1.90 ± 0.09	1.70 ± 0.06	2.85 ± 0.05	1.70 ± 0.10	1.90 ± 0.11
28	22	2.87 ± 0.05	1.60 ± 0.05	2.00 ± 0.05	2.76 ± 0.05	1.87 ± 0.05	1.67 ± 0.05	2.82 ± 0.05	1.72 ± 0.05	1.82 ± 0.05
29	20	2.90 ± 0.08	1.60 ± 0.06	2.00 ± 0.06	2.80 ± 0.07	1.90 ± 0.08	1.70 ± 0.05	2.85 ± 0.05	1.75 ± 0.05	1.85 ± 0.05
30	21	2.80 ± 0.08	1.60 ± 0.06	1.80 ± 0.05	3.00 ± 0.05	1.80 ± 0.06	2.20 ± 0.04	2.90 ± 0.07	1.70 ± 0.08	2.00 ± 0.05
31	20	3.96 ± 0.35	2.20 ± 0.10	2.25 ± 0.06	3.85 ± 0.06	1.92 ± 0.31	2.31 ± 0.31	3.90 ± 0.20	2.05 ± 0.20	2.28 ± 0.13
32	20	4.10 ± 0.72	1.65 ± 0.05	1.95 ± 0.15	3.50 ± 0.10	1.50 ± 0.31	2.15 ± 0.05	3.80 ± 0.31	1.58 ± 0.18	2.05 ± 0.05
33	21	3.90 ± 0.10	2.00 ± 0.10	2.35 ± 0.05	4.30 ± 0.20	1.90 ± 0.10	2.45 ± 0.05	4.10 ± 0.15	1.95 ± 0.10	2.40 ± 0.05
34	20	4.40 ± 0.11	2.16 ± 0.25	2.71 ± 0.31	4.26 ± 0.81	2.20 ± 0.10	2.90 ± 0.35	4.34 ± 0.36	2.18 ± 0.13	2.64 ± 0.33
35	20	3.88 ± 0.37	2.10 ± 0.25	2.30 ± 0.44	4.10 ± 0.38	2.00 ± 0.12	2.50 ± 0.41	3.86 ± 0.37	2.07 ± 0.19	2.63 ± 0.43
36	20	4.39 ± 0.57	2.07 ± 0.19	2.40 ± 0.39	4.23 ± 0.64	2.18 ± 0.19	2.50 ± 0.33	4.31 ± 0.59	2.13 ± 0.16	2.47 ± 0.26
37	21	4.40 ± 0.28	2.50 ± 0.15	3.00 ± 0.34	4.50 ± 0.38	2.00 ± 0.29	2.50 ± 0.21	4.45 ± 0.72	2.25 ± 0.20	2.75 ± 0.33
38	20	4.60 ± 0.37	1.90 ± 0.23	2.70 ± 0.14	4.30 ± 0.25	2.00 ± 0.16	2.30 ± 0.24	4.45 ± 0.69	1.95 ± 0.29	2.50 ± 0.22
39	21	4.75 ± 0.67	1.90 ± 0.10	3.30 ± 0.31	4.50 ± 0.12	1.90 ± 0.18	2.80 ± 0.25	4.60 ± 0.40	1.90 ± 0.24	3.05 ± 0.25
40	18	4.75 ± 0.15	2.05 ± 0.06	2.81 ± 0.20	5.00 ± 0.45	2.80 ± 0.10	2.80 ± 0.05	4.79 ± 0.31	2.38 ± 0.08	2.78 ± 0.13
Total	423	3.74 ± 0.81	1.85 ± 0.37	2.27 ± 0.48	3.66 ± 0.88	1.95 ± 0.33	2.21 ± 0.51	3.70 ± 0.82	1.89 ± 0.32	2.24 ± 0.47

* Freq – Frequency

¶ AP – Anteroposterior

**Relationship between foetal kidney dimensions and GA, maternal weight and maternal height**: There was a very strong significant correlation between foetal renal dimensions and GA (Table 3). The correlation coefficient between GA and mean kidney length, width and anteroposterior diameter were 0.89 (p =0.001), 0.87 (p=0.001) and 0.82 (p=0.001), respectively. A unit change in mean kidney length corresponded to a 79% change in GA (ɼ^2^) showing a very strong association between mean kidney length and GA. There was significant statistical correlation between maternal height and FKL. Maternal weight on the other hand, did not show any significant statistical correlation with FKL (Table 3).

**Table 3:** Correlation between foetal kidney dimensions and GA, maternal weight and maternal height

Kidney parameters	Correlation coefficient (ɼ)	Square of correlation coefficient (ɼ^2^)	p-value
**Gestational age**			
Right kidney length	0.86	0.74	0.001
Right kidney ¶AP diameter	0.79	0.62	0.001
Right kidney width	0.78	0.61	0.001

Left kidney length	0.86	0.74	0.001
Left kidney ¶AP diameter	0.68	0.46	0.001
Left kidney width	0.85	0.72	0.001

Mean kidney length	0.89	0.79	0.001
Mean kidney ¶AP diameter	0.82	0.67	0.001
Mean kidney width	0.87	0.76	0.001

**Maternal weight**			
Right kidney length	-0.02	0.000	0.668
Right kidney ¶AP diameter	0.06	0.004	0.175
Right kidney width	0.12	0.014	0.016

Left kidney length	-0.08	0.006	0.116
Left kidney ¶AP diameter	0.18	0.032	0.001
Left kidney width	-0.04	0.002	0.397

Mean kidney length	-0.05	0.003	0.291
Mean kidney ¶AP diameter	0.13	0.017	0.008
Mean kidney width	0.04	0.002	0.444

**Maternal height**			
Right kidney length	0.15	0.023	0.002
Right kidney ¶AP diameter	0.20	0.040	0.001
Right kidney width	0.22	0.048	0.001

Left kidney length	0.19	0.036	0.001
Left kidney ¶AP diameter	0.09	0.008	0.058
Left kidney width	0.26	0.068	0.001

Mean kidney length	0.18	0.032	0.001
Mean kidney ¶AP diameter	0.16	0.026	0.001
Mean kidney width	0.25	0.063	0.001

¶ AP – Anteroposterior

**Figure 2:**
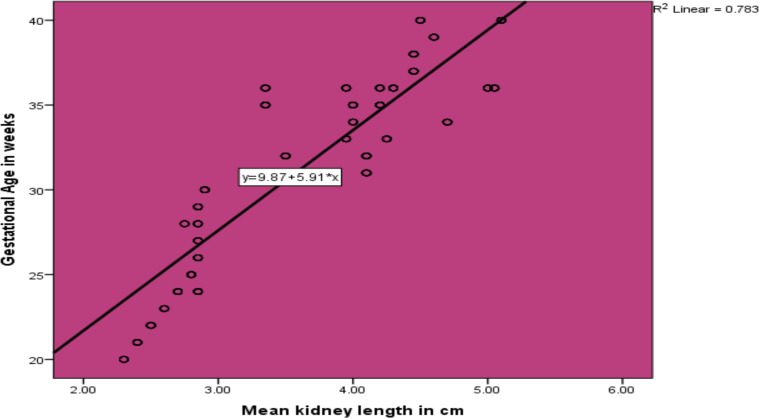
Line graph showing the relationship between GA (in weeks) and mean kidney length (in cm)

**Estimation of GA from mean kidney length**: Figure 2 shows the regression equation: GA = 9.87 + 5.91 x MKL, defining the relationship between mean kidney length and GA. This equation/formula can be used to estimate the GA for a given mean kidney length (Table 4).

**Table 4:** Nomogram showing estimated GA from FKL

Mean kidney length (cm)	Estimated GA	95% CI for estimated GA	

		Minimum	Maximum
2.1	22.3	20.5	24.0
2.2	22.9	21.1	24.7
2.3	23.5	21.7	25.3
2.4	24.1	22.2	25.9
2.5	24.7	22.8	26.5
2.6	25.2	23.3	27.1
2.7	25.8	23.9	27.8
2.8	26.4	24.5	28.4
2.9	27.0	25.0	29.0
3.0	27.6	25.6	29.6
3.1	28.2	26.1	30.3
3.2	28.8	26.7	30.9
3.3	29.4	27.3	31.5
3.4	30.0	27.8	32.1
3.5	30.6	28.4	32.7
3.6	31.2	29.0	33.4
3.7	31.7	29.5	34.0
3.8	32.3	30.1	34.6
3.9	32.9	30.6	35.2
4.0	33.5	31.2	35.8
4.1	34.1	31.8	36.5
4.2	34.7	32.3	37.1
4.3	35.3	32.9	37.7
4.4	35.9	33.4	38.3
4.5	36.5	34.0	38.9
4.6	37.1	34.6	39.6
4.7	37.7	35.1	40.2
4.8	38.2	35.7	40.8
4.9	38.8	36.2	41.4
5.0	39.4	36.8	42.1

## Discussion

The advent and introduction of USS in clinical practice have improved patient evaluation, early diagnosis, treatment, monitoring and prognosis of many clinical conditions. It is a good tool for estimating GA and evaluating growth and abnormalities associated with the foetal kidneys. Despite the fact that the kidney size is affected by growth variations, which appear to mainly affect the anteroposterior and transverse diameters, the kidney length appears to be more stable. This makes it a suitable foetal biometric parameter for the estimation of GA.

This study revealed a mean maternal age ± SD of 29.4 ± 5.6 years, with an age range of 16 – 40 years. These correspond with the reproductive age-group, and are in consonance with the reports of other authors within and outside Nigeria (5,13–15). The index study revealed a significant statistical correlation between maternal height and FKL. This is in contrast with the observations of Edevbie *et al.* (13), Cohen *et al.* (16) and Nahid *et al.* (17), where there was no significant correlation between maternal height and FKL. Maternal weight on the other hand, did not show any significant statistical correlation with FKL. This observation is in agreement with those of Cohen *et al.* (16) and Nahid *et al.* (17), but in contrast with that of Edevbie *et al.* (13), which reported a weak positive correlation between the two variables. The reasons for these variations may not be readily understood; however, the GA at which the anthropometric measurements were taken, the type of study conducted, the sample size and the sample population may have played a role.

Our study revealed a linear increase in FKL with increasing GA. A unit change in mean kidney length corresponded to a 79% change in GA (ɼ^2^). This correlation is in tandem with the reports of other authors (4,5,7,10,13,14,17–19). This finding supports the fact that FKL can be used for the estimation of GA. When pregnant women present or book late, unsure of their last menstrual periods, the obstetrician is saddled with the responsibility of estimating the GA, in order to date pregnancy and subsequently prevent poor foeto-maternal outcomes. In early pregnancy, GA is usually estimated accurately by using the gestational sac diameter and CRL, and later in pregnancy, other foetal biometric parameters (BPD, HC, FL, AC) become more appropriate. Researches have suggested that FKL is more accurate in estimating GA compared to the other biometric indices in the second half of pregnancy (4,7,13,20–22).

This study revealed that the right FKL (3.74 ± 0.81 cm) was slightly longer than the left FKL (3.66 ± 0.88 cm). This finding is in contrast with the reports of Abonyi *et al.* (4), Toosi and Rezaie-Delui (22) and Fitzsimons (23), where the left FKL was longer than the right FKL. However, Konje *et al.* (7) and Kansaria and Parulekar(21) found no significant difference in the lengths of the right and left kidneys. The differential lengths of the right and left kidneys may be due to the differences in genetic makeup and socioeconomic class of people around the globe (24,25).

A nomogram showing the relationship between FKL and EGA was produced from this research. This includes the reference ranges from 20 weeks' GA (kidney length of 2.1 cm) to 40 weeks (kidney length of 5.0 cm). These reference ranges are very similar to the values reported from other centres within Nigeria (4,5,13). However, reference ranges from outside Nigeria were lower than those obtained from our study (19,26,27). This indicates that the reference ranges for other populations may not be reliable for our local population. Therefore, every region should have its own indigenous chart to be used for its local population.

This study is limited by the fact that it is regional-based, and its findings and reference ranges may not reflect those obtainable from other regions of the world. Some of our patients were obese. Obesity limits the optimal visualisation of the kidneys and subsequent assessment of their lengths. However, with our high-resolution ultrasound scan machine, we were able to surmount this limitation. This limitation notwithstanding, our study provides crucial data on the relationship between FKL and GA in normal pregnancy among a sample of pregnant women in our region. The strength of our paper lies in the fact that it is a multicentre study. Ultrasound scans were performed by only four Consultant Radiologists (one for each centre), with special interest and expertise in foetal renal scans, limiting inter-observer differences, and increasing the reliability of our findings.

In conclusion, our study revealed a strong correlation between FKL and EGA. The FKL increases with advancing gestation. The FKL can therefore be used to estimate GA. The nomogram from this research will serve as a reference chart for our local population.

## Figures and Tables

**Figure 1:**
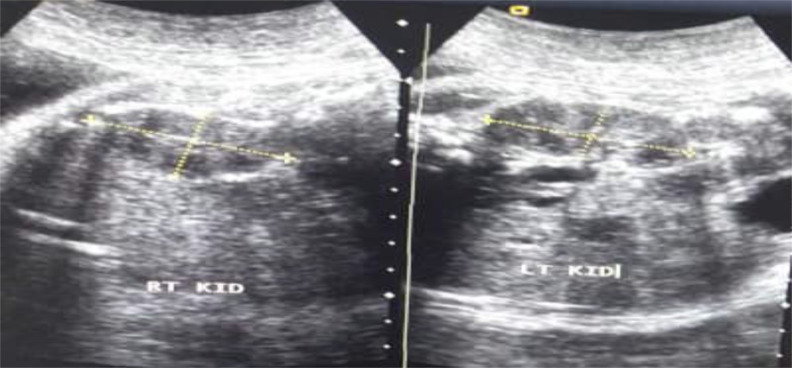
Sonogram of foetal kidney lengths and widths measurements (yellow dotted lines) at 39 weeks' gestational age; Right kidney length = 4.76 cm, Right kidney width = 2.42 cm; Left kidney length = 4.45 cm, Left kidney width = 2.20 cm
